# National trends and characteristics of inpatient detoxification for drug use disorders in the United States

**DOI:** 10.1186/s12889-018-5982-8

**Published:** 2018-08-29

**Authors:** He Zhu, Li-Tzy Wu

**Affiliations:** 1 Department of Psychiatry and Behavioral Sciences, School of Medicine, Duke University Medical Center, BOX 3903, Durham, NC 27710 USA; 2 Department of Medicine, Division of General Internal Medicine, Duke University Medical Center, Durham, NC USA; 3 Duke Clinical Research Institute, Duke University Medical Center, Durham, NC USA; 4 Center for Child and Family Policy, Sanford School of Public Policy, Duke University, Durham, NC USA

**Keywords:** Drug use disorder, Opioid use disorder, Detoxification, Rehabilitation, Inpatient

## Abstract

**Background:**

Prior studies indicate that the opportunity from detoxification to engage in subsequent drug use disorder (DUD) treatment may be missed. This study examined national trends and characteristics of inpatient detoxification for DUDs and explored factors associated with receiving DUD treatment (i.e., inpatient drug detoxification plus rehabilitation) and discharges against medical advice (DAMA).

**Methods:**

We analyzed inpatient hospitalization data involving the drug detoxification procedure for patients aged≥12 years (*n* = 271,403) in the 2003–2011 Nationwide Inpatient Samples. We compared the estimated rate and characteristics of inpatient drug-detoxification hospitalizations between 2003 and 2011 and determined demographic and clinical correlates of inpatient drug detoxification plus rehabilitation (versus detoxification-only) and DAMA (versus transfer to further treatment).

**Results:**

There was no significant yearly change in the population rate of inpatient drug-detoxification hospitalizations during 2003–2011. The majority of inpatient drug detoxification were patients aged 35–64 years, males, and those on Medicaid. Among inpatient drug-detoxification hospitalizations, only 13% received detoxification plus rehabilitation during inpatient care, and up to 14% were DAMA; the most commonly identified diagnoses were opioid use disorder (OUD; 75%) and non-addiction mental health disorders (48%). Being on Medicaid (vs. having private insurance) and having OUD (vs. no OUD) were associated with decreased odds of receiving detoxification plus rehabilitation, as well as increased odds of DAMA.

**Conclusions:**

These findings suggest the presence of a potentially large detoxification-treatment gap for inpatient detoxification patients. They highlight the need for implementing DUD services to improve engagement in receiving further DUD treatment in order to improve recovery and health outcomes.

**Electronic supplementary material:**

The online version of this article (10.1186/s12889-018-5982-8) contains supplementary material, which is available to authorized users.

## Background

Drug detoxification, known as medically managed withdrawal, is a medical intervention for withdrawal symptoms associated with the reduction or the stop of drug use [1, 2]. According to the Treatment Episode Data Set, about 18% of treatment admissions for problem drug users aged≥12 years received detoxification (excluding medication-assisted opioid therapy) in the United States in 2014 [3]. Drug detoxification can only help manage acute withdrawals, and the continuous drug use disorder (DUD) treatments (e.g., rehabilitation, individualized drug counseling, or pharmacotherapy) are needed to achieve a longer-term abstinence or recovery [1, 4]. Although drug detoxification itself is not considered sufficient for treatment, it often offers an initial contact opportunity to engage problem drug users into further DUD treatment [4, 5]. It is critical but challenging to establish effective linkages from initial detoxification to subsequent office-based or follow-up DUD treatment [6–8]. Detoxification occurs in various medical settings. In contrast to outpatient detoxification, inpatient detoxification provides intensive but costly medical care, which may tend to manage patients with serious complications or high-risk, under-treated drug users [9, 10]. There are limited data about the utilization of inpatient drug detoxification from a large sample size. This study seeks to examine trends and characteristics of inpatient detoxification for DUD and related treatment utilization to inform efforts aimed at improving DUD treatment engagement and health outcomes for problem drug users.

First, there are limited data about national trends in inpatient detoxification, especially for DUD. An earlier study found that, between 1992 and 1997, the number of hospital alcohol/drug detoxification discharges in the United States increased 11%, and most of these detoxifications were among those aged 18–44 years, with private insurance, reporting low-income, or having primary alcohol dependence diagnosis [11]. However, similar to the majority of studies on detoxification, alcohol and drug use related detoxification treatments were aggregated into one group in the analysis. There is a lack of data specific for DUD related detoxification. Of note, the prevalence of alcohol use disorder (AUD) has decreased from 7.5% in 2003 to 6.5% in 2011 in the United States [12], while the prevalence of problem drug use has increased. For example, the age-adjusted rate of drug overdose deaths nearly tripled nationwide between 1999 and 2015 [13]; in 2015, there were 52,404 drug overdose deaths, and 63% of these deaths were opioid-related overdose deaths [14]. Opioid-related overdose has become a national crisis [15–17]. Recent data also suggest that cocaine-related overdoses have increased [13]. The earlier data from a convenience sample of a hospital-based alcohol/drug detoxification unit showed that the common primary substances identified among patients were alcohol, cocaine, and opiates [18]. The treatment for problem drug use may differ from that for problem alcohol use on patient demographic and clinical characteristics [2, 3]. The analysis of pooled alcohol and drug detoxification data can obscure the difference in treatment gaps between problem alcohol users and problem drug users.

Second, few data are available on exploring the utilization of inpatient treatment for DUD at the same time with inpatient detoxification. Previous studies have reported a low prevalence of treatment utilization in the samples of drug users from the general population and/or detoxification patients [6, 12, 19]. Recent national survey data showed that only 13% of adults with past-year DUD received any treatment or help for drug use related treatment in the United States [20], and only 19% of detoxification patients started recovery treatment within 30 days in the state of Delaware in 2006 [21]. Besides detoxification, inpatient units can provide intensive medical supervision, urgent pharmaceutical intervention, or rehabilitation treatment for DUD in order to prevent relapse, as well as treatment for comorbid conditions [22–25]. It was found that there was a higher rate of detoxification completion in inpatient settings than in outpatient ones [26]. Unfortunately, some data indicated a decreasing trend in the proportion of patients who received rehabilitation utilization among inpatient drug/alcohol detoxifications (39% in 1992 vs. 21% in 1997) [11]. Male sex, Hispanic ethnicity (vs. whites), unemployment, and lower visit cost were found to be associated with lower odds of receiving treatment for DUD after detoxification [7, 22, 27–29].

Further, patients may choose to leave the treatment before physician recommends discharge, which is defined as discharges against medical advice (DAMA) [30]. Prior data suggest a relatively high DAMA rates among detoxification patients, and DAMA patients with DUD tended to receive inadequate drug treatment [3, 31]. The prevalence of DAMA among substance users who left detoxification ranged from 13 to 52% in a variety of previous studies between 1996 and 2010 [32]. Specifically, unemployment and injection drug use were found to be associated with increased odds of DAMA [32]. A case-control study found that Latino ethnicity and being on Medicaid/no-insurance were associated with elevated odds of DAMA in an inpatient alcohol/drug detoxification sample (*n* = 517 patients), and patients were more likely to be DAMA on Friday or Saturday than Sunday to Thursday [33]. By analyzing the data from 12 states in 2003, Mark et al. [24] found that about 20% of detoxification unit discharges were considered DAMA for inpatients with a primary substance use disorder (SUD) diagnosis. DAMA is an understudied area for DUD. The study of DAMA in inpatient detoxification patients will provide useful predictor information to inform prevention and intervention efforts aimed at reducing the rate of leaving the treatment prematurely and the receipt of insufficient DUD care. Given the clinical implications for DAMA, we also examine the rate and correlates of DAMA.

Detoxification-only without treatment is a clinical concern, as patients receiving detoxification-only without DUD treatment were found to have an increased likelihood of having further drug overdoses and detoxification readmissions [28, 34]. There is a clear need to understand factors associated with further DUD treatment initiation following drug detoxification in order to inform efforts aimed at increasing DUD treatment entry and engagement [1, 5, 7, 35]. To fill these research gaps, this study examines: (1) national trends in the population-based rate and demographic characteristics of inpatient drug detoxification; (2) trends in clinical characteristics of inpatient drug detoxification; (3) factors associated with the receipt of inpatient drug detoxification plus rehabilitation (vs. inpatient drug detoxification-only); and (4) factors associated with DAMA.

## Methods

### Data source

The data of inpatient detoxification were obtained from the Nationwide Inpatient Sample (NIS) of the Healthcare Cost and Utilization Project (HCUP) sponsored by the Agency for Healthcare Research and Quality [36]. The NIS is a 20% stratified sample of U.S. community hospitals drawn from the HCUP State Inpatient Databases, and it is the largest publicly available all-payer inpatient database in the United States [36]. The American Hospital Association (AHA) defined community hospitals as “all nonfederal, short-term general, and special hospitals, including special children’s hospitals, whose facilities and services are available to the public” [37]. The analysis of this study was based on data from 2003 to 2011. In 2012, HCUP changed the NIS’s sampling designs that constrained the analysis of pooling 2003–2011 data with 2012 and later data.

### Study sample

This study focused on non-maternal/non-neonatal inpatient hospitalizations involving drug detoxification procedure for patients aged≥12 years. We studied hospitalization episode data rather than patient-level data because of the nature of the data source. The treatment procedure of drug detoxification was identified through International Classification of Diseases, Ninth Revision, Clinical Modification (ICD-9-CM) procedure codes: (1) drug detoxification (94.65); (2) drug rehabilitation and detoxification (94.66); (3) combined alcohol and drug detoxification (94.68); and (4) combined alcohol and drug rehabilitation and detoxification (94.69) [38]. Each hospitalization record included up to 15 procedure codes, and an inpatient drug-detoxification hospitalization was defined that a hospitalization included at least one above-defined ICD-9-CM drug detoxification procedure code. From 2003 to 2011, there were 271,403 (sample size, unweighted) inpatient drug-detoxification hospitalizations for patients aged≥12 years, and 96% of them received detoxification service as a primary treatment procedure.

### Study variables

#### Inpatient drug detoxification-only and detoxification plus rehabilitation

Based on the ICD-9-CM detoxification procedure codes, we categorized detoxification and treatment during hospital inpatient care into two categories: (1) drug detoxification-only was defined as including ICD-9-CM 94.65 (drug detoxification) or/and 94.68 (combined alcohol and drug detoxification); (2) drug detoxification plus rehabilitation was defined as including ICD-9-CM 94.66 (drug rehabilitation and detoxification) or/and 94.69 (combined alcohol and drug rehabilitation and detoxification).

#### DAMA, routine discharge, and transfer to further treatment

The discharge status after detoxification was identified by the patients’ disposition record in the NIS, and it included DAMA (patients left against medical advice or discontinued care), routine discharge (patients were discharged to home or self-care), and transfer to further treatment (patients were transferred to short-term hospitals, other health care settings [e.g., skilled nursing facilitates, intermediate care facilities], or received home health care). Death during hospitalization and unknown discharge were not reported because of a small sample size (*N* < 10) required by the HCUP.

#### Diagnosis of substance use and mental health disorders

We used ICD-9-CM diagnosis codes to identify substance use and mental health disorders [38]. The first-listed diagnosis was primary diagnosis as a chiefly responsible condition for patient’s admission to inpatient care, and other-listed diagnosis was secondary diagnosis as the pre-existing comorbidity at admission or newly diagnosis during hospitalization. DUD diagnoses included cannabis (304.3×, 305.2×), opioids (including heroin; 304.0×, 304.7×, 305,5×), sedative, hypnotic or anxiolytic (304.1×, 305.4×), stimulant (amphetamines 304.4×, 305.7×; cocaine 304.2×, 305.6×), drug withdrawal (292.0), and other drugs (292.xx [excluding 292.0], 304.5×, 304.6×, 304.8×, 304.9×, 305.3×, 305.8×, 305.9×). AUD diagnoses included ICD-9-CM codes of 291.xx, 303.xx, 305.0×, 357.5, 425.5, 535.3×, and 571.0–571.3. Non-addiction mental health disorder (MHD) diagnoses consist of mood disorders, adjustment disorders, anxiety disorders, schizophrenic, psychotic and delusional disorders, personality disorders, and impulse control and disruptive behavior disorders (See Additional file 1).

#### Demographic and hospital characteristics

Among inpatient drug-detoxification hospitalizations, we examined patient’s age at admission, sex, race/ethnicity (non-Hispanic white, non-Hispanic black, Hispanic, Asian/Pacific-Islander/Native-American, non-Hispanic other race, and unknown), median household income for patient’s ZIP code (lowest, 2nd, 3rd, and highest quartile), and primary expected payer (Medicare, Medicaid, private, self-pay, other payers). The unknown race/ethnicity referred to cases from the states not providing racial/ethnical information in certain years, and we have coded them into one group because they were not randomly missing (*n* = 45,292) [36]. The household income increased with higher quartile value, and income range for quartile varied by year [36]. Other payers included no charge, worker’s compensation, CHAMPUS, CHAMPVA, Title V, and other government programs. Hospital characteristics included census of hospital region (Northeast, Midwest, South and West) and hospital location (rural and urban).

### Data analyses

Descriptive statistics of the estimated annual inpatient drug-detoxification hospitalization rate per million population aged≥12 years were used to identify the detoxification trend. We compared the demographic and clinical characteristics of inpatient drug detoxification, and chi-square and t-test analyses were used to detect the differences for categorical and continuous variables between 2003 and 2011, respectively. Logistic regressions were conducted to determine demographic and clinical correlates of hospital DUD treatment (inpatient drug detoxification plus rehabilitation vs. inpatient drug detoxification-only) and discharge status (DAMA vs. transfer to further treatment; DAMA vs. routine discharge). Because of a large sample size, we used a significance level *p* < 0.01 to reduce potential false-positive results [39]. All analyses were performed in Stata 13.1 (StataCorp, College Station, TX) using survey commends to account for NIS sampling design [40].

## Results

### Trends and demographic characteristics of inpatient drug-detoxification hospitalizations (Fig. 1 and Table 1)

Overall, the estimated population rate of inpatient drug-detoxification hospitalizations was stable during the study period. In 2011, the estimated rate was 500 inpatient drug-detoxification hospitalizations per million population aged≥12 years compared to 529 in 2003, but this decrease was not statistically significant (See Additional file 1).

**Fig. 1:**
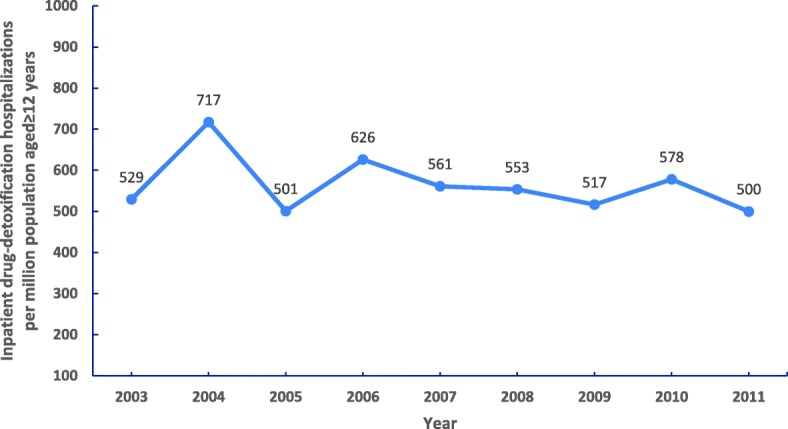
The estimated annual inpatient drug-detoxification hospitalization rate per million population aged ≥12 years: 2003–2011 Nationwide Inpatient Samples

**Table 1:** Demographic and hospital characteristics of inpatient drug-detoxification hospitalizations for patients aged≥12 years: 2003–2011 Nationwide Inpatient Samples

Year	Overall (2003–2011)		2003		2011		*p* value^‡^
Sample size, unweighted N	271,403		27,626		28,692		
Weighted N	1,284,276		128,277		131,371		
Weighted column, %	%	(95% CI)	%	(95% CI)	%	(95% CI)	
Mean of age, years	38.79	38.29–39.29	37.24	35.91–38.57	39.94	38.17–41.71	**0.017**
Age group, years							**< 0.001**
12–17	0.73	(0.45–1.18)	1.19	(0.57–2.48)	0.53	(0.17–1.63)	
18–25	13.95	(12.82–15.17)	14.94	(11.16–19.72)	14.89	(11.65–18.83)	
26–34	22.53	(21.54–23.54)	24.92	(22.90–27.05)	23.02	(19.46–27.01)	
35–49	45.01	(43.45–46.58)	46.74	(42.32–51.20)	36.99	(33.03–41.13)	
50–64	16.24	(15.21–17.32)	10.93	(9.21–12.92)	22.17	(18.38–26.50)	
65+	1.55	(1.36–1.76)	1.29	(0.83–1.99)	2.41	(1.71–3.37)	
Sex							0.286
Male	65.27	(63.97–66.55)	63.02	(60.03–65.92)	64.82	(61.52–67.98)	
Female	34.60	(33.34–35.89)	36.69	(33.85–39.62)	35.10	(31.96–38.38)	
Race/Ethnicity							**0.001**
White, non-Hispanic	44.77	(41.18–48.41)	44.57	(34.68–54.90)	53.98	(42.42–65.13)	
Black, non-Hispanic	20.74	(17.78–24.05)	14.58	(9.80–21.14)	27.19	(17.69–39.36)	
Hispanic	9.63	(7.69–12.00)	8.13	(5.10–12.74)	7.60	(5.03–11.33)	
Asian/Pacific-Islander/Native-American	0.67	(0.52–0.87)	0.34	(0.21–0.55)	0.99	(0.47–2.10)	
Other races, non-Hispanic	3.85	(2.80–5.27)	4.30	(1.59–11.11)	3.87	(1.81–8.09)	
Unknown^a^	16.78	(13.46-20.72)	23.28	(12.83–38.47)	4.40	(1.72–10.77)	
Household income							0.731
Lowest quartile	36.41	(33.55–39.36)	33.25	(25.96–41.43)	33.51	(26.23–41.67)	
2nd quartile	20.75	(19.46–22.11)	24.98	(21.17–29.23)	21.09	(17.64–25.00)	
3rd quartile	18.72	(17.28–20.26)	20.27	(16.26–24.96)	20.60	(17.47–24.13)	
Highest quartile	17.66	(15.96–19.49)	17.14	(13.29–21.82)	19.39	(15.16–24.47)	
Primary expected payer							0.219
Medicare	11.89	(11.10–12.74)	11.19	(8.45–14.67)	14.02	(10.85–17.92)	
Medicaid	39.89	(36.03–43.87)	28.68	(19.99–39.28)	40.37	(30.77–50.76)	
Private	22.72	(20.44–25.18)	28.86	(21.43–37.64)	21.74	(15.95–28.91)	
Self-pay	18.53	(15.78–21.62)	19.65	(11.64–31.22)	17.66	(10.19–28.85)	
Other payers	6.76	(5.48–8.32)	11.52	(6.34–20.02)	6.04	(3.48–10.30)	
Hospital region							0.793
Northeast	44.02	(38.66–49.51)	41.70	(26.61–58.53)	36.80	(23.99–51.78)	
Midwest	20.44	(16.44–25.12)	20.71	(11.21–35.08)	18.15	(8.62–34.25)	
South	25.02	(21.12–29.38)	22.87	(14.38–34.35)	31.51	(20.13–45.64)	
West	10.52	(8.60–12.81)	14.72	(8.65–23.92)	13.54	(7.25–23.90)	
Hospital location							0.126
Rural	6.99	(5.36–9.07)	10.81	(4.90–22.17)	5.05	(2.70–9.27)	
Urban	92.88	(90.80–94.52)	89.19	(77.83–95.10)	94.83	(90.61–97.22)	
Detoxification procedure array							0.817
Primary procedure	96.48	(95.74–97.10)	96.57	(94.85–97.73)	96.83	(94.65–98.14)	
Secondary procedure	3.52	(2.90–4.26)	3.43	(2.27–5.15)	3.17	(1.86–5.35)	
Detoxification and treatment							0.157
Drug detoxification-only	87.45	(84.58–89.85)	79.82	(68.08–88.01)	88.47	(79.32–93.88)	
Drug detoxification plus rehabilitation	12.55	(10.15–15.42)	20.18	(11.99–31.92)	11.53	(6.12–20.68)	
Discharge status^b^							**0.007**
Routine discharge	76.65	(75.23–78.00)	79.06	(76.04–81.80)	75.01	(69.62–79.72)	
Transfer to further treatment	9.44	(8.41–10.57)	6.79	(4.92–9.30)	12.57	(8.91–17.44)	
Discharges against medical advice (DAMA)	13.67	(12.69–14.72)	13.62	(11.29–16.33)	12.30	(10.00–15.05)	

*CI* confidence interval

^‡^We reduced the significant level to 0.01 due to a relatively large sample. Boldface: *p* < 0.01

^a^Race/ethnicity information were not available in some states in some years, which were coded into unknown category

^b^The categories of died and other discharges were not reported due to small sample size (less than 10)

Among inpatient drug-detoxification hospitalizations for patients aged≥12 years, 61% were aged 35–64 years, 65% were males, 45% were non-Hispanic whites, 36% were residents in the area with lowest household income, 40% reported Medicaid as the primary payer, 44% resided in the northeast region, and 93% were treated in urban hospitals. Notably, between 2003 and 2011, there was a significant increase in the proportion of inpatient drug detoxification for patients aged 50–64 years (11% in 2003 vs. 22% in 2011; *p* < 0.001), but a decrease in the proportion for patients aged 35–49 years (47% in 2003 vs. 37% in 2011; *p* = 0.002).

### Trends in clinical characteristics of inpatient drug-detoxification hospitalizations (Table 2)

Among inpatient drug-detoxification hospitalizations for patients aged≥12 years, 71, 12 and 11% were admitted to hospitalization primarily for any DUD diagnosis, AUD diagnosis and any non-addiction MHD diagnosis, respectively. The most common DUDs as primary diagnoses were drug withdrawal (34%) and opioid use disorder (OUD; 27%). There was no significant change in DUDs, AUDs, and MHDs as primary diagnoses between 2003 and 2011, but significant increases in the proportions of inpatient drug detoxification were observed for the majority of DUDs and non-addiction MHDs as secondary diagnoses, including any DUD, any non-addiction MHD, OUDs, sedative use disorders, mood disorders, schizophrenic/psychotic/delusional disorders, and anxiety disorders.

**Table 2:** Clinical characteristics of inpatient drug-detoxification hospitalizations for patients aged≥12 years: 2003–2011 Nationwide Inpatient Samples

Year	Overall (2003–2011)		2003		2011		*p* value^‡^
Sample size, unweighted N	271,403		27,626		28,692		
Weighted column, %	%	(95% CI)	%	(95% CI)	%	(95% CI)	
Any-listed diagnosis							
Any drug use disorder	99.13	(98.96–99.28)	99.12	(98.82–99.35)	99.18	(98.71–99.48)	0.811
Drug withdrawal	47.45	(42.85–52.09)	34.71	(23.76–47.56)	52.67	(41.57–63.52)	0.037
Opioid use disorders	74.83	(72.70–76.85)	72.37	(63.93–79.47)	75.60	(68.64–81.43)	0.529
Stimulant use disorders^a^	30.06	(27.63–32.61)	25.27	(19.71–31.79)	28.62	(22.89–35.14)	0.447
Sedative use disorders	13.21	(11.99–14.52)	**9.65**	**(7.27–12.69)**	**17.74**	**(13.28–23.31)**	**0.003**
Cannabis use disorders	12.14	(11.09–13.28)	10.19	(7.40–13.89)	14.26	(11.48–17.57)	0.081
Other drug-related disorders^b^	11.87	(10.75–13.08)	12.90	(9.83–16.76)	11.08	(8.66–14.07)	0.409
Alcohol use disorders	42.37	(39.41–45.38)	35.68	(29.05–42.90)	41.32	(32.54–50.69)	0.334
Any non-addiction mental health disorder	47.93	(45.29–50.59)	**43.16**	**(35.30–51.38)**	**58.98**	**(51.82–65.78)**	**0.004**
Mood disorders	38.35	(36.08–40.67)	34.66	(28.25–41.67)	46.42	(40.39–52.55)	0.013
Schizophrenic/psychotic/ delusional disorders	3.94	(3.58–4.32)	3.35	(2.49–4.48)	5.19	(3.74–7.15)	0.048
Anxiety disorders	10.82	(9.89–11.81)	**8.17**	**(6.53–10.18)**	**17.38**	**(13.90–21.53)**	**< 0.001**
Adjustment disorders	0.95	(0.79–1.13)	1.09	(0.79–1.50)	1.26	(0.51–3.08)	0.761
Personality/impulse-control/disruptive behavior disorders	7.12	(6.29–8.04)	7.39	(5.55–9.78)	7.81	(5.74–10.54)	0.795
Primary diagnosis							
Any drug use disorder	71.26	(68.79–73.61)	71.73	(64.93–77.67)	64.53	(55.84–72.35)	0.175
Drug withdrawal	33.68	(29.28–38.38)	24.60	(14.85–37.89)	35.37	(24.36–48.19)	0.214
Opioid use disorders	27.27	(24.12–30.66)	36.95	(25.52–50.06)	20.51	(15.07–27.29)	0.013
Stimulant use disorders^a^	5.45	(3.95–7.48)	5.16	(3.38–7.80)	4.38	(2.18–8.61)	0.688
Sedative use disorders	1.38	(1.15–1.67)	1.21	(0.85–1.71)	1.68	(1.05–2.68)	0.266
Cannabis use disorders	0.34	(0.23–0.49)	0.54	(0.18–1.61)	0.31	(0.12–0.76)	0.443
Other drug-related disorders^b^	3.14	(2.68–3.67)	3.27	(2.39–4.47)	2.28	(1.63–3.18)	0.119
Alcohol use disorders	12.45	(11.07–13.96)	11.53	(8.66–15.20)	15.70	(10.32–23.16)	0.223
Any non-addiction mental health disorder	11.07	(9.60–12.72)	11.83	(8.68–15.92)	15.08	(9.90–22.80)	0.351
Mood disorders	9.22	(8.00–10.60)	9.67	(7.03–13.16)	12.43	(8.37–18.05)	0.323
Schizophrenic/psychotic/ delusional disorders	1.21	(1.01–1.45)	1.41	(0.95–2.09)	1.37	(0.82–2.26)	0.922
Anxiety disorders	0.26	(0.19–0.36)	0.29	(0.15–0.59)	0.54	(0.16–1.85)	0.391
Adjustment disorders	0.30	(0.20–0.44)	0.37	(0.22–0.62)	0.70	(0.16–2.98)	0.414
Personality/impulse-control/disruptive behavior disorders	0.08	(0.05–0.15)	0.08	(0.04–0.16)	0.05	(0.02–0.14)	0.377
Secondary diagnosis							
Any drug use disorder	80.16	(77.10–82.90)	**68.08**	**(57.56–77.03)**	**87.04**	**(81.71–90.99)**	**< 0.001**
Drug withdrawal	13.77	(11.97–15.80)	10.11	(7.14–14.15)	17.33	(12.10–24.19)	0.030
Opioid use disorders	47.73	(43.88–51.60)	**35.59**	**(25.86–46.67)**	**55.25**	**(45.23–64.86)**	**0.009**
Stimulant use disorders^a^	24.79	(23.05–26.62)	20.41	(16.28–25.27)	24.35	(19.77–29.60)	0.246
Sedative use disorders	11.83	(10.72–13.04)	**8.44**	**(6.25–11.32)**	**16.07**	**(12.13–20.99)**	**0.002**
Cannabis use disorders	11.81	(10.81–12.89)	9.66	(7.15–12.93)	13.95	(11.29–17.12)	0.044
Other drug-related disorders^b^	9.12	(8.30–10.00)	10.04	(7.53–13.26)	9.11	(7.09–11.64)	0.615
Alcohol use disorders	36.12	(33.34–38.99)	29.44	(23.79–35.81)	33.63	(26.96–41.04)	0.375
Any non-addiction mental health disorder	41.38	(39.24–43.55)	**35.40**	**(29.13–42.22)**	**50.67**	**(44.93–56.40)**	**0.001**
Mood disorders	29.74	(28.08–31.45)	**25.47**	**(20.90–30.65)**	**34.82**	**(30.19–39.77)**	**0.008**
Schizophrenic/psychotic/ delusional disorders	2.81	(2.53–3.13)	**1.99**	**(1.47–2.69)**	**4.00**	**(2.75–5.79)**	**0.004**
Anxiety disorders	10.60	(9.70–11.57)	**7.90**	**(6.31–9.85)**	**16.98**	**(13.68–20.88)**	**< 0.001**
Adjustment disorders	0.65	(0.56–0.75)	0.72	(0.48–1.07)	0.57	(0.40–0.80)	0.378
Personality/impulse-control/disruptive behavior disorders	7.07	(6.25–7.98)	7.33	(5.50–9.71)	7.77	(5.71–10.50)	0.785

*CI* confidence interval

^‡^We reduced the significant level to 0.01 due to a relatively large sample. Boldface: The estimate in 2003 in the category differed from the estimate in 2011 (*p* < 0.01)

^a^Stimulant included cocaine and amphetamine

^b^Other drug-related disorders included drugs other than listed in the table defined by ICD-9-CM

Overall, among inpatient drug-detoxification hospitalizations, 99% had any-listed (primary or secondary) DUD diagnosis, 42% had any-listed AUD, and 48% had any-listed non-addiction MHD. It is notable that about three-fourths of inpatient drug-detoxification hospitalizations had any-listed OUD diagnosis. Similarly, increases in the proportion of inpatient drug-detoxification hospitalizations were found for sedative use disorders (10% in 2003 vs. 18% in 2011; *p* = 0.003), any non-addiction MHD (43% in 2003 vs. 59% in 2011; *p* = 0.004), and anxiety disorders (8% in 2003 vs. 17% in 2011; *p* < 0.001).

### Inpatient drug detoxification plus rehabilitation vs. detoxification-only (Table 3)

Between 2003 and 2011, only 13% of inpatient drug-detoxification hospitalizations received inpatient drug detoxification plus rehabilitation. Compared with drug detoxification-only hospitalizations, detoxification plus rehabilitation hospitalizations had higher proportions of ages 12–25 years, females, non-Hispanic whites, persons on Medicare or private insurance, residents in the west areas, and those with MHD diagnoses, respectively. For example, those on private insurance accounted for 41% of detoxification plus rehabilitation hospitalizations whereas only 20% of detoxification-only hospitalizations.

**Table 3:** Characteristics and adjusted odds ratios of inpatient drug detoxification plus rehabilitation vs. detoxificantion-only among inpatient drug-detoxification hospitalizations for patients aged≥12 years: 2003–2011 Nationwide Inpatient Samples

Drug treatment	Drug detoxification-only		Drug detoxification plus rehabilitation		Drug detoxification plus rehabilitation (1) vs. drug detoxification-only (0)		
Sample size, unweighted N (weighted row %)	236,682 (87.45%)		34,721 (12.55%)		242,476^b^		
Weighted column %/Adjusted odds ratio (AOR)	%	(95% CI)	%	(95% CI)	AOR	(95% CI)	*p* value^‡^
Age group, years							
12–17	0.52	(0.34–0.78)	2.22	(1.00–4.86)	**2.72**	**(1.43–5.17)**	**0.002**
18–25	13.29	(12.12–14.56)	18.56	(16.31–21.04)	1.00		
26–34	22.33	(21.28–23.41)	23.89	(21.93–25.96)	0.88	(0.79–0.98)	0.015
35–49	46.06	(44.45–47.68)	37.69	(34.65–40.82)	**0.70**	**(0.62–0.78)**	**< 0.001**
50–64	16.40	(15.28–17.58)	15.12	(13.53–16.86)	**0.67**	**(0.58–0.78)**	**< 0.001**
65+	1.41	(1.27–1.56)	2.53	(1.78–3.59)	0.75	(0.52–1.08)	0.118
Sex							
Male	65.90	(64.50–67.27)	60.91	(58.66–63.12)	1.00		
Female	34.00	(32.64–35.39)	38.80	(36.63–41.02)	1.02	(0.95–1.09)	0.601
Race/Ethnicity							
White, non-Hispanic	42.85	(39.13–46.64)	58.15	(50.62–65.32)	1.00		
Black, non-Hispanic	22.29	(19.08–25.87)	9.96	(7.20–13.62)	**0.63**	**(0.46–0.87)**	**0.005**
Hispanic	10.41	(8.25–13.06)	4.21	(2.77–6.35)	**0.48**	**(0.33–0.71)**	**< 0.001**
Asian/Pacific-Islander/Native-American	0.66	(0.49–0.87)	0.79	(0.49–1.30)	0.65	(0.38–1.11)	0.113
Other races, non-Hispanic	4.18	(3.00–5.79)	1.53	(1.10–2.12)	**0.50**	**(0.31–0.81)**	**0.005**
Unknown^a^	16.44	(12.98-20.61)	19.11	(12.2–28.65)	0.84	(0.48–1.48)	0.557
Household income							
Lowest quartile	37.30	(34.26–40.45)	30.15	(24.52–36.45)	1.00		
2nd quartile	20.77	(19.38–22.23)	20.63	(17.90–23.65)	0.90	(0.74–1.09)	0.272
3rd quartile	18.13	(16.59–19.78)	22.87	(20.26–25.70)	1.11	(0.91–1.35)	0.308
Highest quartile	16.87	(15.15–18.75)	23.14	(18.76–28.19)	1.09	(0.85–1.41)	0.493
Primary expected payer							
Medicare	10.94	(10.21–11.72)	18.53	(15.54–21.94)	1.07	(0.83–1.36)	0.612
Medicaid	42.63	(38.50–46.87)	20.74	(14.79–28.29)	**0.42**	**(0.27–0.64)**	**< 0.001**
Private	20.05	(17.90–22.38)	41.38	(35.29–47.74)	1.00		
Self-pay	19.23	(16.18–22.70)	13.61	(9.58–18.97)	**0.40**	**(0.25–0.64)**	**< 0.001**
Other payers	6.93	(5.51–8.69)	5.57	(4.20–7.34)	**0.40**	**(0.27–0.59)**	**< 0.001**
Hospital region							
Northeast	46.28	(40.45–52.20)	28.27	(18.61–40.45)	1.00		
Midwest	21.01	(16.65–26.15)	16.47	(10.95–24.02)	1.02	(0.49–2.12)	0.968
South	24.16	(19.99–28.88)	31.05	(22.29–41.40)	1.42	(0.77–2.63)	0.261
West	8.56	(6.91–10.55)	24.21	(16.53–34.01)	2.52	(1.17–5.47)	0.019
Hospital location							
Rural	5.57	(4.30–7.19)	16.90	(9.88–27.41)	1.00		
Urban	94.33	(92.71–95.61)	82.75	(72.28–89.82)	**0.32**	**(0.18–0.58)**	**< 0.001**
Any-listed alcohol use disorder							
No	57.98	(54.61–61.28)	55.23	(50.92–59.47)	1.00		
Yes	42.02	(38.72–45.39)	44.77	(40.53–49.08)	**1.30**	**(1.13–1.50)**	**< 0.001**
Any-listed opioid use disorder							
No	23.97	(21.85–26.22)	33.56	(29.04–38.40)	1.00		
Yes	76.03	(73.78–78.15)	66.44	(61.60–70.96)	**0.75**	**(0.63–0.89)**	**0.001**
Any-listed any non-addiction mental health disorder^c^							
No	53.78	(51.01–56.53)	40.12	(34.69–45.80)	1.00		
Yes	46.22	(43.47–48.99)	59.88	(54.20–65.31)	1.18	(0.94–1.48)	0.149

*AOR* adjusted odds ratio, *CI* confidence interval

^‡^We reduced the significant level to 0.01 due to a relatively large sample. Boldface: *p* < 0.01

^a^Race/ethnicity information were not available in some states in some years, which were coded into unknown category

^b^Hospitalizations with missing values or zero trend weight were excluded. The regression model included all variables listed in the first column and controlled for survey year

^c^Any mental health disorder included adjustment disorders, anxiety disorders, attention-deficit, conduct, and disruptive behavior disorders, impulse control disorders, mood disorders, personality disorders, schizophrenia, psychotic, delusional disorders

We conducted adjusted logistic regression analyses to examine factors associated with receiving inpatient drug rehabilitation with detoxification. The adjusted analysis indicated that ages 12–17 (vs. ages 18–25), non-Hispanic whites (vs. non-Hispanic blacks), having private insurance (vs. being on Medicaid), rural hospital (vs. urban), AUD diagnosis (vs. no), and no any-listed OUD diagnosis (vs. OUD) were associated with increased odds of receiving hospital drug detoxification plus rehabilitation vs. detoxification-only.

### DAMA vs. routine and transfer discharges (Table 4)

Generally, there was no significant change in the distribution of discharge status for inpatient drug detoxification between 2003 and 2011. Among inpatient drug-detoxification hospitalizations, 77% were discharged routinely, 14% were DAMA, and only 9% were transferred to further Drug treatment. Adjusted logistic regressions found that ages 18-25 years (vs. ≥35), males (vs. females), Medicaid (vs. private insurance), OUD (vs. no OUD), and no any non-addiction MHD (vs. MHD) were associated with elevated odds of DAMA (vs. transfer to further treatment) and DAMA (vs. routine discharge).

**Table 4:** Characteristics and adjusted odds ratio of discharge against medical advice (DAMA), routine, and transfer discharges among inpatient drug-detoxification hospitalizations for patients aged≥12 years: 2003–2011 Nationwide Inpatient Samples

Detoxification discharge status	DAMA		Routine		Transfer to further treatment		DAMA (1) vs. routine (0)			DAMA (1) vs. transfer to further treatment (0)		
Sample size, unweighted N (weighted row %)	36,885 (13.67%)		208,291 (76.65%)		25,566 (9.44%)		219,536^b^			54,832^b^		
Weighted column %/Adjusted odds ratio (AOR)	%	(95% CI)	%	(95% CI)	%	(95% CI)	AOR	(95% CI)	*p* value^‡^	AOR	(95% CI)	*p* value^‡^
Age group, years												
12–17	0.25	(0.16–0.40)	0.79	(0.47–1.34)	0.91	(0.59–1.42)	**0.39**	**(0.26–0.57)**	**< 0.001**	**0.34**	**(0.19–0.61)**	**< 0.001**
18–25	16.16	(14.42–18.07)	13.56	(12.41–14.79)	13.82	(12.51–15.24)	1.00			1.00		
26–34	27.20	(25.96–28.48)	21.94	(20.86–23.05)	20.51	(19.45–21.62)	0.98	(0.93–1.03)	0.469	1.06	(0.98–1.16)	0.157
35–49	44.65	(42.43–46.88)	45.30	(43.68–46.94)	43.39	(41.34–45.46)	**0.72**	**(0.67–0.78)**	**< 0.001**	**0.85**	**(0.76–0.94)**	**0.003**
50–64	11.17	(10.10–12.34)	16.89	(15.77–18.08)	18.17	(16.99–19.42)	**0.53**	**(0.48–0.58)**	**< 0.001**	**0.56**	**(0.49–0.63)**	**< 0.001**
65+	0.57	(0.45–0.71)	1.51	(1.32–1.73)	3.19	(2.64–3.84)	**0.33**	**(0.27–0.40)**	**< 0.001**	**0.18**	**(0.14–0.24)**	**< 0.001**
Sex												
Male	70.99	(69.35–72.57)	64.30	(62.99–65.58)	64.91	(63.01–66.76)						
Female	28.87	(27.29–30.49)	35.58	(34.31–36.87)	35.00	(33.16–36.89)	**0.88**	**(0.85–0.91)**	**< 0.001**	**0.85**	**(0.80–0.91)**	**< 0.001**
Race/Ethnicity												
White, non-Hispanic	43.23	(38.42–48.18)	44.48	(40.77–48.24)	48.73	(44.72–52.77)	1.00			1.00		
Black, non-Hispanic	19.98	(16.75–23.66)	20.94	(17.70–24.61)	20.54	(17.31–24.20)	0.84	(0.74–0.96)	0.011	1.03	(0.85–1.25)	0.762
Hispanic	13.15	(9.70–17.60)	8.97	(7.18–11.17)	9.95	(7.86–12.53)	0.98	(0.86–1.12)	0.758	1.11	(0.83–1.47)	0.481
Asian/Pacific-Islander/Native-American	0.63	(0.46–0.86)	0.68	(0.52–0.90)	0.65	(0.50–0.85)	1.02	(0.79–1.32)	0.851	1.37	(1.01–1.85)	0.044
Other races, non-Hispanic	5.52	(3.68–8.20)	3.54	(2.56–4.87)	3.95	(2.87–5.40)	1.07	(0.88–1.31)	0.490	1.25	(0.95–1.66)	0.112
Unknown^a^	14.21	(10.74-18.57)	17.81	(14.20–22.10)	12.39	(9.66–15.76)	1.07	(0.88–1.31)	0.485	1.24	(0.90–1.69)	0.183
Household income												
Lowest quartile	37.70	(33.71–41.87)	36.93	(34.05–39.92)	30.66	(27.26–34.28)	1.00			1.00		
2nd quartile	19.81	(17.95–21.82)	20.90	(19.65–22.21)	21.03	(18.68–23.59)	1.00	(0.93–1.07)	0.938	0.79	(0.65–0.95)	0.012
3rd quartile	17.03	(15.28–18.93)	18.65	(17.32–20.06)	21.62	(18.24–25.43)	0.93	(0.86–1.00)	0.055	**0.66**	**(0.51–0.86)**	**0.002**
Highest quartile	16.94	(14.34–19.91)	17.68	(15.99–19.50)	18.14	(15.99–20.50)	**0.90**	**(0.81–1.00)**	**0.060**	**0.78**	**(0.66–0.93)**	**0.006**
Primary expected payer												
Medicare	9.08	(8.21–10.04)	11.98	(11.20–12.80)	15.13	(13.36–17.09)	**1.62**	**(1.50–1.75)**	**< 0.001**	**1.38**	**(1.20–1.59)**	**< 0.001**
Medicaid	47.27	(42.00–52.60)	38.61	(34.70–42.67)	39.92	(35.29–44.74)	**1.64**	**(1.49–1.81)**	**< 0.001**	**1.60**	**(1.33–1.91)**	**< 0.001**
Private	14.85	(12.75–17.24)	23.89	(21.46–26.50)	24.35	(21.30–27.68)	1.00			1.00		
Self-pay	22.08	(18.33–26.35)	18.67	(15.78–21.96)	12.34	(10.76–14.13)	**1.68**	**(1.49–1.89)**	**< 0.001**	**2.43**	**(1.94–3.06)**	**< 0.001**
Other payers	6.57	(4.74–9.04)	6.62	(5.41–8.07)	8.07	(5.81–11.12)	**1.70**	**(1.35–2.13)**	**< 0.001**	0.99	(0.74–1.33)	0.954
Hospital region												
Northeast	58.49	(51.60–65.06)	40.51	(35.16–46.09)	51.11	(44.61–57.58)	1.00			1.00		
Midwest	16.41	(12.45–21.31)	22.04	(17.65–27.17)	13.67	(10.98–16.88)	**0.60**	**(0.50–0.72)**	**< 0.001**	1.30	(0.89–1.90)	0.180
South	17.01	(12.68–22.43)	27.08	(22.91–31.69)	20.37	(16.55–24.79)	**0.53**	**(0.44–0.63)**	**< 0.001**	1.01	(0.72–1.41)	0.967
West	8.10	(6.25–10.44)	10.37	(8.47–12.64)	14.86	(11.11–19.59)	**0.77**	**(0.65–0.92)**	**0.004**	0.78	(0.57–1.06)	0.115
Hospital location												
Rural	5.87	(3.93–8.67)	7.01	(5.36–9.14)	8.57	(6.20–11.72)	1.00			1.00		
Urban	94.10	(91.30–96.04)	92.84	(90.71–94.50)	91.34	(88.19–93.71)	1.04	(0.81–1.33)	0.768	**1.75**	**(1.28–2.40)**	**< 0.001**
Any-listed alcohol use disorder												
No	55.93	(51.94–59.85)	59.05	(55.91–62.12)	48.59	(45.67–51.51)	1.00			1.00		
Yes	44.07	(40.15–48.06)	40.95	(37.88–44.09)	51.41	(48.49–54.33)	**1.13**	**(1.06–1.21)**	**< 0.001**	**0.83**	**(0.75–0.92)**	**< 0.001**
Any-listed opioid use disorder												
No	18.92	(16.60–21.47)	25.36	(23.21–27.63)	32.61	(29.82–35.53)	1.00			1.00		
Yes	81.08	(78.53–83.40)	74.64	(72.37–76.79)	67.39	(64.47–70.18)	**1.33**	**(1.23–1.45)**	**< 0.001**	**1.73**	**(1.49–2.02)**	**< 0.001**
Any-listed any non-addiction mental health disorder^c^												
No	67.58	(65.03–70.03)	50.19	(47.39–52.99)	45.03	(41.95–48.14)	1.00			1.00		
Yes	32.42	(29.97–34.97)	49.81	(47.01–52.61)	54.97	(51.86–58.05)	**0.58**	**(0.54–0.62)**	**< 0.001**	**0.46**	**(0.42–0.51)**	**< 0.001**

*DAMA* discharges against medical advice, *AOR* adjusted odds ratio, *CI* confidence interval

^**‡**^We reduced the significant level to 0.01 due to a relatively large sample. Boldface: *p* < 0.01

^a^Race/ethnicity information were not available in some states in some years, which were coded into unknown category

^b^Hospitalizations with missing values or zero trend weight were excluded. The regression model included all variables listed in the first column and controlled for survey year

^c^Any mental health disorder included adjustment disorders, anxiety disorders, attention-deficit, conduct, and disruptive behavior disorders, impulse control disorders, mood disorders, personality disorders, schizophrenia, psychotic, delusional disorders

## Discussion

Patients with severe DUD and/or comorbid medical complications may have a high likelihood of admitting to hospital inpatient care [2, 41]. Inpatient settings provide a unique opportunity to engage those with DUD into treatment. This study extends previous research by examining national trends in inpatient detoxification for DUD and factors associated with potentially inadequate treatment. Our findings imply that more efforts are needed to improve engagement for receiving follow-up DUD treatment to prevent relapse and to treat comorbid medical/mental disorders in order to facilitate recovery. First, there was a relatively stable trend in the rate of inpatient drug detoxification between 2003 and 2011, and most of inpatient hospitalizations for drug detoxification were found among those aged 35–64 years, males, non-Hispanic whites, residents in the low-income area, or those on Medicaid. Second, the two most commonly identified diagnoses among inpatient detoxification hospitalizations were OUD (75%) and any non-addiction MHD (48%). Third, only 13% of inpatient hospitalizations for drug detoxification also received hospital rehabilitation, and up to 14% were DAMA. Fourth, being on Medicaid (vs. having private insurance) and having OUD (vs. no OUD) were associated with lower odds of receiving inpatient drug detoxification plus rehabilitation and elevated odds of DAMA. Overall, the findings suggest the presence of a large treatment gap for DUD, and the need to improve the further use of DUD care following the receipt of brief or episodic detoxification treatments.

Our findings highlight the concern that only a small proportion of hospitalized patients receiving detoxification appeared to have received additional DUD treatment during their inpatient care. The finding of a very low prevalence of receiving rehabilitation during inpatient detoxification was consistent with previous studies. Mark et al. [11] found that 21% of inpatient alcohol/drug detoxifications received inpatient rehabilitation in a national sample. This study provides newer national-level estimates for inpatient drug detoxification, and our results suggest a similarly low level of rehabilitation service use during inpatient detoxification. The low utilization of additional DUD treatment during inpatient detoxification may be related to a high hospital treatment cost and/or a lack of an infrastructure or organizational support to promote DUD treatment [10, 42, 43]. Since the 1980s, some health plans (e.g., managed care) have sought to reduce the cost of DUD treatment by shifting inpatient care toward outpatient care [44]. Other factors that may influence the receipt of additional DUD services in the inpatient units include the severity of patients’ DUD, health insurance status, and the hospital-related patient placement criteria [1, 45]. Recent findings suggested that, after the Affordable Care Act (ACA) Medicaid expansion, there was an increase in having Medicaid and using Medicaid to pay treatment among patients admitted to SUD specialty treatment in the expansion states in the United States [46]. The extension of health insurance may improve treatment opportunities and financial security for problem drug users.

Moreover, our results suggest a low prevalence of receiving further DUD treatment (e.g., outpatient treatment) after inpatient drug detoxification. Only 9% of those with inpatient drug detoxification in this sample were transferred at discharge, and up to 14% were considered as DAMA. They demonstrate a need to identify barriers (e.g., patient, health system, financial factors) to receiving further DUD treatments and to develop effective strategies to link the detoxification patients with outpatient or specialty treatment services for improving the continuity of DUD care [7, 43]. Acevedo et al. [42] indicated that the financial incentives or electronic reminders to medical agencies had an effect on increasing treatment use after detoxification among residential facilities. Ford and Zarate [41] reported that the implementation of a comprehensive service model (including case management, assessment at admission, and post-detoxification follow-up requirement etc.) increased the enrollment rate of follow-up treatment after inpatient detoxification to reach 71% within 1 year, along with other positive outcomes (e.g., increased employment and decreased arrest rates). Spear [47] suggested the use of inter-organizational networks at SUD treatment settings was associated with an increase in the continuity of treatment after detoxification and a decrease in the rate of detoxification readmissions. Additionally, consistent with prior research, our findings suggest that the provision of additional referral or linkage services should be offered to detoxification patients with Medicaid to reduce DAMA and to increase treatment retention [7, 32, 33, 48]. The redesign of the Medicaid-funded SUD treatment systems, such as providing comprehensive services of publicly funded SUD care and expanding SUD services through increasing insurance coverage to low-income population, could help improve access to treatment for people on Medicaid [49, 50].

Another notable concern is that OUD accounted for 75% of all inpatient drug detoxification examined in this study, but those with OUD showed a low likelihood of receiving inpatient drug detoxification plus rehabilitation and a high likelihood of DAMA. Opioid overdose deaths are an epidemic in the United States [51]. Opioid overdose death rate per 100,000 population increased approximately 200% between 2000 and 2014 [14]. Both the number of intensive care unit admissions for patients with opioid overdoses and the mortality rate of these overdoes patients showed a significant increase between 2009 and 2015 [52]. The nation survey data suggested that only 19% of persons aged ≥12 years with past-year OUD received any opioid-specific treatment in the past year [19]. Thus, a low likelihood of those with OUD receiving inpatient drug detoxification plus rehabilitation and a high likelihood of DAMA reinforce a high need to target persons with OUD to receive proper and timely DUD treatment [53]. Adults with OUD may take 7–10 years (on average) to remit from opioid misuse [54]. Treatment for OUD requires coordinated care to address medical comorbidities and to help maintain continuity of care to reduce morbidity and mortality [55–57]. One clinical trial found that patients in the group of receiving the linkage service of hospitalization to follow-up office-based opioid agonist treatment (OAT) were more likely to enter and stay in the OAT than those without receiving the linkage service [23]. Therefore, early detection for opioid misuse and engagement of people with OUD who received detoxification into medication assisted treatment (MAT) or other office-based DUD care will be important to reduce OUD problems and mitigate the treatment gap [35, 54].

Our findings also emphasize the need to treat comorbid MHDs to prevent relapse and improve treatment effectiveness and retention [8, 58, 59]. MHDs were common among persons with DUD, according to the National Survey of Substance Abuse Treatment Services (N-SSATS), 48% of clients in the SUD treatment were diagnosed as having a comorbidity of MHD [60]. Similarly, this study found that 59% of inpatient drug detoxification had a diagnosis of MHDs (e.g., mood, anxiety, personality, schizophrenic disorders) in 2011 with an increasing pattern since 2003. The finding of an increase in the share of comorbid DUD/MHD patients among inpatient drug detoxification may reflect a higher severity level of the inpatient sample (such as comorbidity) as well as a lack of coordinated care to treat comorbid diagnoses. For example, the National Survey on Drug use and Health (NSDUH) data showed that only 8.5% of adults with SUD and MHD received treatment for two disorders at a specialty facility in 2015 [12]. In addition, insurance coverage affects the access to behavioral health care. The managed health plans may tend to limit the use of inpatient care in order to control the cost [44]. The co-occurring MHDs and DUDs without timely treatment could worsen their clinical courses and increase the overall healthcare costs or resource utilization [61, 62]. Treatment barriers to DUDs and comorbid MHDs may include personal financial concerns, stigma, low motivation or perception for treatment need, and a lack of specialized or coordinated care services [63, 64]. Correspondingly, expanding insurance coverage for behavioral health services (e.g., increasing reimbursement for pharmacotherapy), integrating coordinated care networks (e.g., reducing referral delay), and developing feasible treatment models for combined mental and SUD problems may help improve enrollment and effectiveness for treating comorbid SUD and mental disorders [53, 58, 59]. In addition, screening and assessment of comorbid MHDs for patients with DUD at admission and making timely referrals to specialty mental health providers can be useful in improving access to treatment.

### Limitation

These findings should be interpreted within the context of study limitations. First, the NIS datasets do not include information about SUD and psychiatric treatment facilities, which may underestimate detoxification treatment use. Second, the NIS data represent hospital discharge encounter data, and they do not allow to identify patient-level readmissions. Third, the identification of clinical characteristics and rehabilitation treatment was based on ICD-9-CM codes in the NIS dataset. Thus, these findings on treatment use were conservative or may be underestimated because treatments outside of the studied facility were not available for analysis. Four, some inpatient drug detoxification were assigned ICD-9-CM drug withdrawal code without specific DUD diagnosis or with more than one DUD diagnosis. Fifth, the unknown race/ethnicity accounted for about 17% of the study sample, which may affect the results. For example, the hospitalization data from Minnesota, Ohio, and West Virginia in the NIS datasets have no racial information, and these states include a higher proportion of non-Hispanic whites that the national average [65]. However, the HCUP conducted a comparison of demographic distributions among the US population, the NIS sample, and the National Hospital Discharge Survey. The racial composition of the NIS sample was found to be generally similar to those from the U.S. population [36]. Finally, this analysis cannot capture the most recent information about inpatient drug detoxification. Due to substantial changes in the study design of the NIS in 2012, we only analyzed 2003–2011 data to study the national trends and clinical characteristics of inpatient detoxification (including DAMA). However, to our knowledge, this study is among the first of its kind to examine the trend data in these years for DUD specific detoxification characteristics. Prior studies were conducted over a decade ago and cannot provide DUD specific information [11]. This study used the largest national sample of inpatient detoxification available to provide newer national-level estimates for DUD to inform inpatient-based DUD service efforts. More studies are needed to monitor the most recent trends in DUD detoxification and treatment, particularly, within the context of changes in DUD treatment (e.g., expanding the provision of MAT for OUD and other SUDs).

## Conclusions

Prevalence and characteristics of inpatient detoxification and DAMA have been understudied, as typical national survey data, such as NSDUH and NSEARC, cannot provide adequate information for the analysis reported in this study [12, 20]. Our findings from the largest national inpatient sample indicate that there was a potentially large gap in engaging detoxification patients with DUD into subsequent DUD treatment, including patients with OUD. The growing concerns of the opioid overdose epidemic and an increasing proportion of comorbid DUDs and MHDs found in this national sample reinforce the need to increase clinical efforts to engage patients with OUD during detoxification into medication-assisted treatment or other DUD treatment to prevent relapse and facilitate faster remission. More efforts are needed to ensure the effective linkage between initial treatment admission for problem drug use to more formal treatment for DUD across various medical settings.

## Additional file

Additional file 1: **Table S1.** Definitions of substance use disorder and mental health disorder diagnoses; **Table S2.** The number and population-based rate of inpatient drug-detoxification hospitalizations by year: 2003–2011 Nationwide Inpatient Samples. (DOCX 15 kb)

## Abbreviations

AHA: American Hospital Association
AUD: Alcohol use disorder
CHAMPUS: Civilian Health and Medical Program of the Uniformed Services
CHAMPVA: Civilian Health and Medical Program of the Department of Veterans Affairs
DAMA: Discharges against medical advice
DUD: Drug use disorder
HCUP: Healthcare Cost and Utilization Project
ICD-9-CM: International Classification of Diseases, Ninth Revision, Clinical Modification
MAT: Medication-Assisted Treatment
MHD: Mental health disorder
NIS: Nationwide Inpatient Sample
NSDUH: National Survey on Drug use and Health
NSEARC: National Epidemiologic Survey on Alcohol and Related Conditions
N-SSATS: National Survey of Substance Abuse Treatment Services
OAT: Opioid agonist treatment
OUD: Opioid use disorder
SUD: Substance use disorder
