# Supplementary material for: National trends and characteristics of inpatient detoxification for drug use disorders in the United States
Source: BMC Public Health. 2018 Aug 29;18:1073. doi: 10.1186/s12889-018-5982-8 (PMC6114033; doi:10.1186/s12889-018-5982-8)
Supplement: Supplementary file 1 — Table S1. Definitions of substance use disorder and mental health disorder diagnoses; Table S2. The number and population-based rate of inpatient drug-detoxification hospitalizations by year: 2003–2011 Nationwide Inpatient Samples. (DOCX 15 kb) [file 12889_2018_5982_MOESM1_ESM.docx]

**National trends and characteristics of inpatient detoxification for drug use disorders in the United States**

He Zhu, Li-Tzy Wu

**Table S1. Definitions of substance use disorder and mental health disorder diagnoses**

| **Diagnoses** | **International Classification of Diseases, Ninth Revision, Clinical Modification (ICD-9-CM) codes** |
| --- | --- |
| Drug withdrawal | 292.0 |
| Opioid/heroin-related disorders | 304.0x; 304.7x; 305.5x |
| Sedative use disorders | 304.1x; 305.4x |
| Stimulant use disorders (cocaine and amphetamine) | 304.2x; 305.6x; 304.4x;305.7x |
| Cannabis use disorders | 304.3x;305.2x |
| Other drug-related disorders | 292.xx (excluding 292.0); 304.5x; 304.6x; 304.8x; 304.9x; 305.3x; 305.8x; 305.9x |
| Alcohol use disorders | 291.xx, 303.xx, 305.0x, 357.5; 425.5; 535.3x; 571.0-571.3 |
| Non-addiction mental health disorders | Mood disorders: 293.83; 296.xx; 300.4; 301.13; 311  Anxiety disorders: 293.84; 300.0x; 300.2x; 300.3; 300.5; 308.x; 309.81; 313.0; 313.1; 313.21; 313.22  Schizophrenic, psychotic, delusional disorders: 293.81; 293.82; 295.xx; 297.xx; 298.xx  Adjustment disorders: 309.0; 309.2x; 309.3; 309.4; 309.8x; 309.9  Personality disorders: 301.xx (excluding 301.13)  Impulse-control, disruptive behavior: 312.xx; 314.xx; 313.81 |

**Table S2. The number and population-based rate of inpatient drug-detoxification hospitalizations by year: 2003-2011 Nationwide Inpatient Samples**

| **Year** | **Unweighted N** | **Weighted N** | **95%CI** | **Rate per million population aged≥12 years** | **95%CI** |
| --- | --- | --- | --- | --- | --- |
| **2003** | 27,626 | 128,277 | (88,620-167,933) | 529 | (366-693) |
| **2004** | 37,780 | 175,867 | (120,299-231,435) | 717 | (491-944) |
| **2005** | 26,487 | 124,170 | (90,321-158,019) | 501 | (364-637) |
| **2006** | 33,252 | 156,978 | (98,205-215,750) | 626 | (392-861) |
| **2007** | 29,423 | 142,102 | (102,578-181,626) | 561 | (405-717) |
| **2008** | 30,129 | 141,536 | (93,598-189,473) | 553 | (366-741) |
| **2009** | 26,703 | 133,386 | (91,330-175,442) | 517 | (354-680) |
| **2010** | 31,311 | 150,591 | (102,903-198,278) | 578 | (395-761) |
| **2011** | 28,692 | 131,371 | (93,264-169,477) | 500 | (355-645) |

CI: confidence interval.
